# Experimental Study on Dynamic Splitting Characteristics of Carbon Fiber Reinforced Concrete

**DOI:** 10.3390/ma14010094

**Published:** 2020-12-28

**Authors:** Zhan-Yang Chen, Jun Yang

**Affiliations:** State Key Laboratory of Explosion Science and Technology, Beijing Institute of Technology, Beijing 100081, China; yangj@bit.edu.cn

**Keywords:** SHPB, CFRC, splitting tensile strength, failure patterns

## Abstract

Due to the non-uniform tension and compression strength of concrete, carbon fiber can be added to concrete to improve its static tensile behavior and increase the tension–compression ratio. In view of the destructive consequences of impacts and explosions, it is necessary to study the dynamic responses of carbon fiber reinforced concrete (CFRC) structures. Therefore, the effects of the stress rates and carbon fiber contents on the dynamic tension behavior of CFRC were investigated in this paper. The dynamic splitting tests of concrete with the fiber contents of 0, 0.1, 0.2, and 0.3% were carried out by using a split Hopkinson pressure bar (SHPB) device with a diameter of 74 mm. We found that with the increase of fiber content, the static tensile strength of CFRC increases obviously, but the increased amplitude tends to decrease. The dynamic tensile strength and dynamic increase factor (DIF) both increase with the increase of stress rate, but the growth rate slows down, showing an obvious rate effect. The rate sensitivity of ordinary concrete is higher than CFRC. There are significant differences in the influence of carbon fiber on the dynamic and static strength of concrete. In the design of concrete mixing proportion, the content of carbon fiber should be appropriately selected to meet the requirements of dynamic and static mechanical properties.

## 1. Introduction

Concrete is by far the most important building material. Due to its relatively low price, simple production, and wide range of application, its consumption is increasing all over the world. The requirements of concrete strength and mechanical properties have gradually increased with the rapid development of high-rise buildings and long-span buildings. The damage to a concrete structure is often affected by its tensile properties. In the uniaxial compression, the damage to concrete is essentially controlled by the tensile damage perpendicular to the direction of pressure [1]. However, concrete generally has inherent defects of low tensile strength, poor toughness, and uneven tensile and compressive strength. The structure will inevitably be affected by impact, explosion and other ultimate load during application. The response characteristics of the structure under impact loading are different from those under static loading, and the uneven distribution of internal stress in the structure will be further aggravated, which will bring serious hidden dangers to the structure [2,3]. Therefore, the mechanical properties of concrete under the high strain rate have attracted more and more attention, and effective measures to improve them must be considered by researchers. Incorporating concrete into fibers is an effective way to solve the problem of low tensile strength and poor ductility [4,5,6], which can increase the energy absorption capacity of concrete and provide a more plastic structure. Fibers are mainly made of steel, carbon or polymers.

The steel fibers and polymer fibers are easily obtained and incorporated into the concrete matrix, which can effectively inhibit the initiation and expansion of cracks during the destruction of concrete structures, and improve durability and tensile strength, so they have become widely used fibers [7,8,9,10]. Therefore, many researchers have focused on steel fiber and polymer fiber reinforced concrete and conducted a lot of research on its static and dynamic mechanical properties [11,12,13,14,15,16,17]. However, compared with other fibers, carbon fiber has many potential advantages, including a series of excellent properties such as high strength, high modulus, high-temperature resistance, corrosion resistance, light weight, electrical conductivity, etc. [18]. Compared with ordinary concrete, CFRC has the advantages of high strength, light weight, crack resistance, and shock resistance, so it has considerable application potential in actual engineering.

D.D.L. Chung [19] first found that by adding carbon fibers of a certain size and specification into concrete, the material has the function of self-sensing internal stress, strain and damage. Then, researchers further studied the influence of different surface treatment methods of carbon fiber on the pressure sensitivity and stability of composite materials and improved the bond strength between the carbon fiber and cement matrix through the treatment of silane, ozone, and potassium dichromate [20]. Deng [21] made strain sensors out of carbon fiber cement-based composites, conducted three-point bending tests on prefabricated concrete beams, and studied the relationship between the average strain of concrete beams and the external load. The results indicated that the carbon fiber cement-based composite sensor is sensitive to the strain of the concrete beam, and the strain is linearly dependent on the load. Liu [22] and Manuel [23] studied the electrical conductivity of carbon fiber cement-based composites and indicated that it can be used for intelligent floor heating materials and the repair and reinforcement of cement-based materials under an ultra-low temperature environment. Mastali [24] found that increasing the volume fraction and length of carbon fiber can significantly improve the mechanical properties and impact resistance of concrete. Xiong [25] discussed the effect of carbon fiber content on the mechanical properties and microstructure of CFRC, and considered that the increase of carbon fiber content could significantly improve the ultimate bearing capacity and fracture toughness of CFRC. Zhou [26] studied the effect of short carbon fiber on the mechanical properties and microstructure of high performance concrete (HPC) through the uniform experiments. The results indicated that incorporation of short carbon fibers could improve high brittleness, low tensile strength, and result in far less stress–strain curve of HPC than that of ordinary concrete. Zhou [27] divided the static stress–strain curve of carbon fiber concrete based on the laboratory basic mechanical property test, established the mathematical expressions of the stress–strain curve with different contents, and determined the relationship between corresponding parameters and carbon fiber volume ratio. Giner V.T [28] studied the influence of carbon fiber on the damping ratio and dynamic elastic modulus of concrete containing micro-silicon powder and showed that the dynamic elastic performance of concrete is higher than that of static, and the compressive strength of concrete slightly decreases with the addition of carbon fiber. Zahra [18] compared the blast resistance of conventional concrete panels and long carbon fiber reinforced concrete panels through explosion tests and numerical analysis. The addition of long carbon fibers significantly improved the blast resistance and reduced the cracking degree of concrete panels. It can be seen that there are many researches on the pressure sensitivity, electrothermal effect, and static mechanical properties of CFRC at present, and a relatively mature understanding has been obtained. However, there are few studies on the dynamic properties of CFRC, which need further study. Considering that the tensile strength of CFRC is more severe than the damage of compressive strength, the study of the dynamic splitting tensile performance of CFRC has important engineering and scientific significance.

After a brief introduction to the entire test process, the test results on the static tensile strength of CFRC were reported. The influence of fiber content on the splitting tensile strength and failure patterns of CFRC was investigated by analyzing the test results. Then, considering the action of dynamic loading, a series of dynamic splitting tensile tests were carried out on concrete with different carbon fiber contents using the split Hopkinson pressure bar (SHPB) system. Combined with high-speed imaging technology, the crack propagation process and dynamic tensile properties of CFRC, including failure morphology, stress time-history curve, and dynamic tensile strength, were studied. The effects of carbon fiber content and strain rate on the mechanical properties of CFRC were analyzed. The differences in the mechanical properties of CFRC under static and dynamic loading were discussed based on the change of meso-structure. The test results can provide a reference for practical engineering application and stability analysis of carbon fiber concrete under dynamic load.

## 2. Experimental Procedure and Methods

This section mainly briefly describes the test methods and procedure, including the preparation of materials, the selection of test equipment and the methods of data processing, the preparation process of test pieces, etc.

### 2.1. Materials and Specimens Preparation

**Figure 1:**
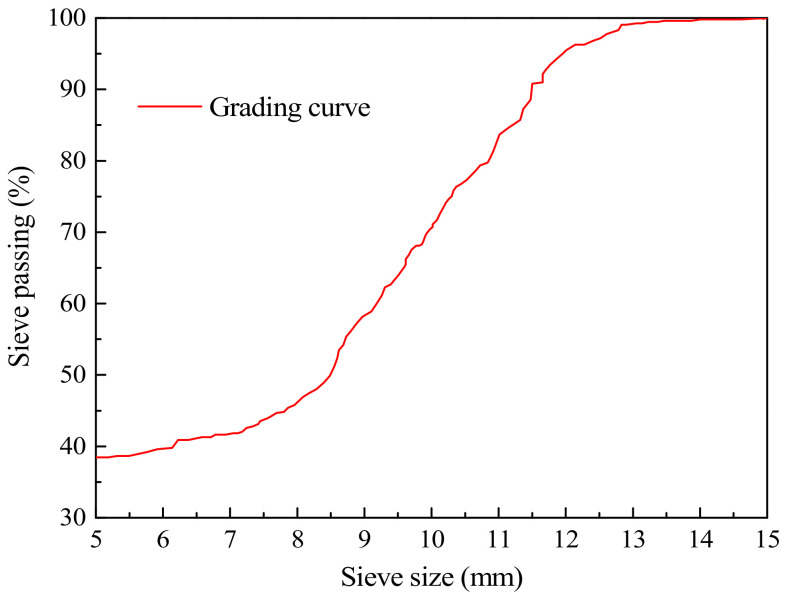
Coarse aggregate grading curve.

Raw materials: The cement was made of ordinary Portland cement (P.O42.5), the silica fume was produced in Sichuan Province, China, and the composition of the cement and silica fume are shown in Table 1. The fine aggregate was river sand with a density of 2600 kg·m^−3^, the maximum particle size of 5 mm, and the fineness modulus of 2.70 was used as fine aggregate. The water used in the test was tap water, and the chemical composition is shown in Table 2. The crushed stone with a particle size of 5–15 mm was used for coarse aggregate, and the grading curve is shown in Figure 1. The first-grade fly ash of 1250 mesh and the waste tire rubber powders of 40 mesh were used in the test. The water-reducer was polycarboxylic acid superplasticizer (Type F) (Shengshi building materials, Guangzhou, China) [29]. The test used PAN-based carbon fiber with a diameter of 7 μm, and the relevant parameters of fiber are shown in Table 3. Based on the concrete mix ratio in reference [30], combined with the influence of silica fume, fly ash and other materials, the final mix proportion of CFRC is listed in Table 4. The carbon fiber was added into the concrete matrix to form CFRC by the volume fractions of 0.1, 0.2, and 0.3%. The corresponding specimen types were CFRC0.1, CFRC0.2, and CFRC0.3, respectively. The concrete specimens without carbon fiber were the control group (CG).

**Figure 2:**
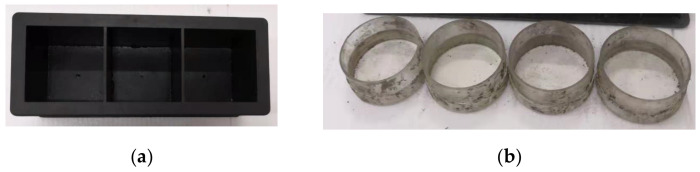
Specimen preparation molds (**a**) cube molds; (**b**) cylinder molds.

Li [32] suggested that when the thickness of the disk specimen is about half of the diameter, the influence of inertia effect on the test results can be eliminated. Combined with previous research work [33,34], in this test the size of the dynamic splitting specimens was a cylinder with a diameter of 70 mm and a height of 35 mm. In order to contrast with the dynamic test, the static tensile test used a specimen of the same size as the dynamic split test. Since the maximum particle size of the aggregate was 15 mm, less than 31.5 mm, the size of the static compressed specimens was 100 mm × 100 mm × 100 mm [35]. The specimens were prepared by the pouring method, pouring the coarse aggregate and fine aggregate into the mixer, mixing for 2 min to make it evenly mixed, then putting in cement and other gel materials, and continuing to mix. The carbon fiber was slowly put into the running mixer, mixed thoroughly to evenly disperse the carbon fiber, water was added and mixed for 3 min. The mixture was put into the cube molds with a side length of 100 mm and the cylinder molds with a diameter of 70 mm and a height of 35 mm (Figure 2). After using the vibration table to consolidate, the specimens were put it into the curing room, and after 24 h were taken out of the molds and tested after standard curing for 28 days [36]. The compressive strength of control group was 60.08 MPa.

### 2.2. Test Methods

#### 2.2.1. Static Test

**Figure 3:**
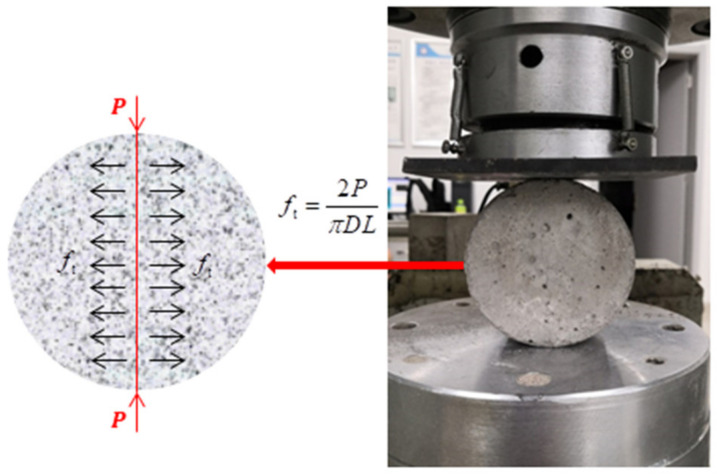
Static splitting test.

At present, the static tensile strength of brittle materials such as concrete and rock are generally determined by direct tensile test and splitting tensile test [37,38]. Because it is difficult to make the axis of the tensile load coincide with the axis of the specimen in the direct tensile test, the experimental data of the direct tensile test are often inaccurate. Then, the Brazilian splitting test was proposed as an alternative tensile test method to make up for the defects of the direct tensile test [14]. Since the strain rate of the static splitting tensile test was relatively low, a rock mechanics testing machine was used for testing, as shown in Figure 3. The test device adopted a 1000 kN force sensor and a 50 mm stroke sensor to measure the vertical force and vertical deformation of the specimen in the vertical direction and utilized a 2.5 mm displacement sensor to measure the lateral deformation of the specimen in two horizontal directions.

During the loading process, the relatively uniform tensile stress perpendicular to the loading direction was formed on the vertical diameter plane, which eventually led to the failure of the specimen along the vertical diameter. Elasticity analysis [39,40] showed that the tensile stress generated on the loading plane in the static splitting test could be determined by Equation (1).

(1) $$f_{t}=\frac{2P}{\pi DL}$$

where *P* is the compressive force applied to the specimen, and *D* and *L* are the specimen diameter and length, respectively.

#### 2.2.2. Dynamic Test

**Figure 4:**
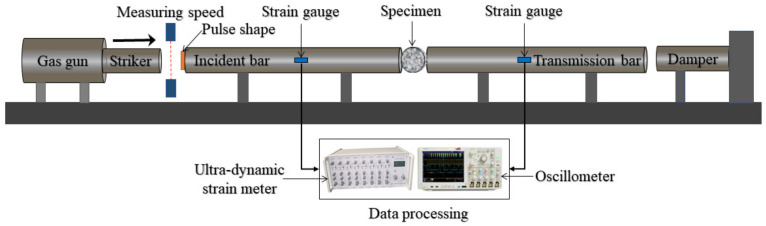
Schematic diagram of split Hopkinson pressure bar (SHPB) test device.

The 74-mm-diameter SHPB test device was used to conduct the dynamic splitting test on concrete specimens. The length of the striker bar, incident bar, and transmission bar were 0.4, 3.20, and 1.80 m, respectively. All the pressure bars of the SHPB device were alloy steel, with a density of 7.8 g·cm^−3^, an elastic modulus of 210 GPa, and a longitudinal wave velocity of 5190 m·s^−1^. The SHPB test device is shown in Figure 4.

In the dynamic test, under the action of driving pressure, the striker bar impacted on the incident bar to generate an incident wave, and propagated along the incident bar. Due to the difference of wave impedance between the specimen and the pressure bar, one part of the incident wave was reflected back to the incident bar and generated a reflected wave, while the other part propagated the transmission bar through the specimen and generated a transmission wave. The super dynamic strain indicator was used to measure incident wave, reflected wave, and transmitted wave in the bar. Then the stress and strain in the bar were calculated according to the propagation theory of the one-dimensional elastic stress wave. The resistance strain gauges with a model of BX120-3AA and a sensitivity coefficient of 2.08 ± 0.01 mm were pasted in the incident bar. In order to acquire weak transmission wave accurately, the semiconductor strain gauges with a resistance of 120 Ω and a sensitivity coefficient of 110 ± 0.05 were pasted in the transmission bar.

The results of Gomez [41] and Guo [42] indicated that when the stress equilibrium was achieved, the dynamic tensile stress distribution in the center of the heterogeneous concrete specimen would be similar to that of homogeneous materials, which could be estimated by static elasticity analysis. Therefore, the data of the SHPB dynamic splitting test can be calculated by Equation (2).

(2) $$\sigma_{t}(t)=\frac{2AE}{\pi DL}\varepsilon_{t}(t)$$

where *σ_t_*(*t*) is the stress of the specimen; *ε_t_*(*t*) is the transmitted strain in the pressure bar; *A* is the cross section area of the pressure bar; *E* is the elastic modulus.

## 3. Test Results and Analysis

### 3.1. Static Test

The tensile strength and failure patterns of concrete specimens with different carbon fiber contents under static load were mainly analyzed in this part. The static test can provide a basis for the discussion and analysis of subsequent dynamic tensile test results. It should be noted that the steel strips were not used to distribute the load between the specimen and the loading plate in this test. It is mainly because of the existence of the steel strips in the dynamic splitting test that the specimen is difficult to be placed, and there is wave reflection at the interface between the steel strip and the test piece, which interferes with the incident wave and reflected wave. The transmission waveguide measured by the strain gauge results in complex and inaccurate data processing [42]. To compare the value of the static and dynamic tensile strength conveniently, the steel strips were not adopted in the static splitting test either.

**Figure 5:**
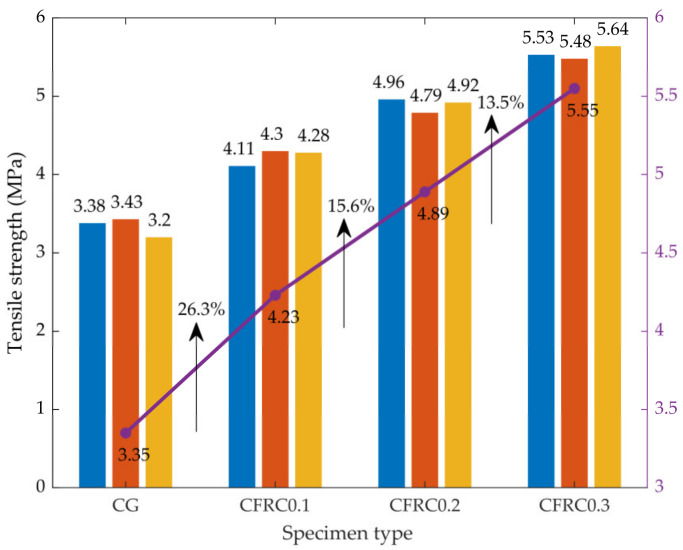
The results of static tensile strength.

Static splitting test loading was carried out in the form of control force, and the loading rate was 0.1 kN·s^−1^. Three parallel specimens were tested for each group, and the static tensile strength of the specimens can be calculated by Equation (1). The tensile strength of concrete with different carbon fiber contents is shown in Figure 5 (the fold line is the average of the results).

As shown in Figure 5, the static tensile strengths of CG, CFRC0.1, CFRC0.2, and CFRC0.3 were 3.35, 4.23, 4.89, and 5.55 MPa, respectively. With the increase of fiber content, the tensile strength increased significantly. Since the elastic modulus of carbon fiber is much higher than that of concrete, under the condition of equal tensile strain, carbon fiber has a constraint effect on concrete, which can delay and prevent the expansion of cracks to a certain extent. When the crack is created, the load of the cracked section acts on the fiber passing through the crack. Fibers are bonded with concrete to transfer the load to the non-cracked concrete matrix, and the static tensile strength of the concrete is significantly improved [26]. At the same time, when the carbon fiber content increased from 0% to 0.3%, the tensile strength increased by 26.3, 15.6 and 13.5 for each increase of 0.1%. It indicated that the increased amplitude of static tensile strength decreased with the increase of fiber content.

**Figure 6:**
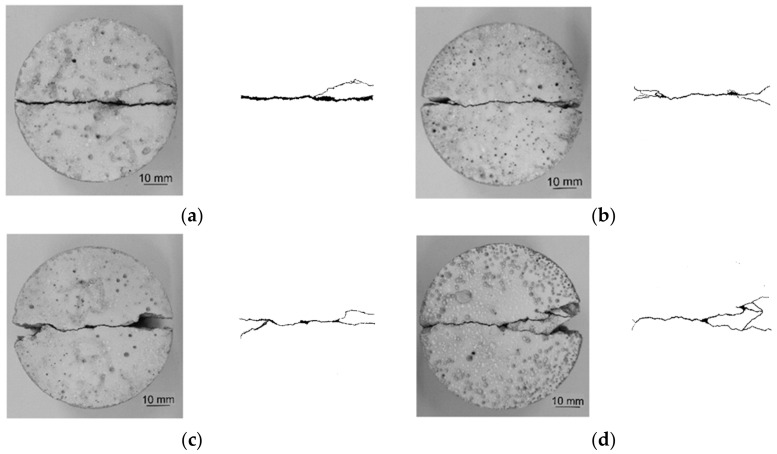
Failure modes of specimens under static loading, (**a**) CG, (**b**) CFRC0.1, (**c**) CFRC0.2, (**d**) CFRC0.3.

The failure patterns of CG, CFRC0.1, CFRC0.2, and CFRC0.3 are shown in Figure 6.

The CG specimen had an obvious straight crack and relatively smooth fracture surfaces. The specimen was split into two semicircles, and the tensile strain capacity was insufficient, showing significant brittle failure characteristics. The main reason was that the water evaporated rapidly in the process of hydration reaction, resulting in many micro-cracks and pores in the matrix. When the specimen was subjected to static loading, stress concentration occurred in the crack tip, and micro-cracks expanded from the center to both ends gradually, eventually forming a straight crack leading to brittle failure of the specimen [14]. With the expansion of the main crack, some secondary micro-cracks and triangular fracture zones were generated on both ends of CFRC0.1 and CFRC0.2, and the micro-cracks at one end of CFRC0.3 increased. Carbon fiber, as a kind of ultrafine fiber, could be used to bridge the cracks in concrete. However, the restraint ability of carbon fiber on the crack propagation of concrete was limited, and the specimen still maintained the characteristics of brittle failure. It can be seen that the effect of carbon fiber in the matrix did not significantly improve the deformation capacity of CFRC, although the specimen experienced some lateral displacement during the failure process. In general, the stress generated by the static load was effectively transferred between carbon fiber and concrete matrix, so the tensile strength of carbon fiber could be fully utilized to make up for the lack of tensile strength of the concrete matrix, but the improvement of ductility was not obvious.

### 3.2. Dynamic Splitting Tensile Test

**Figure 7:**
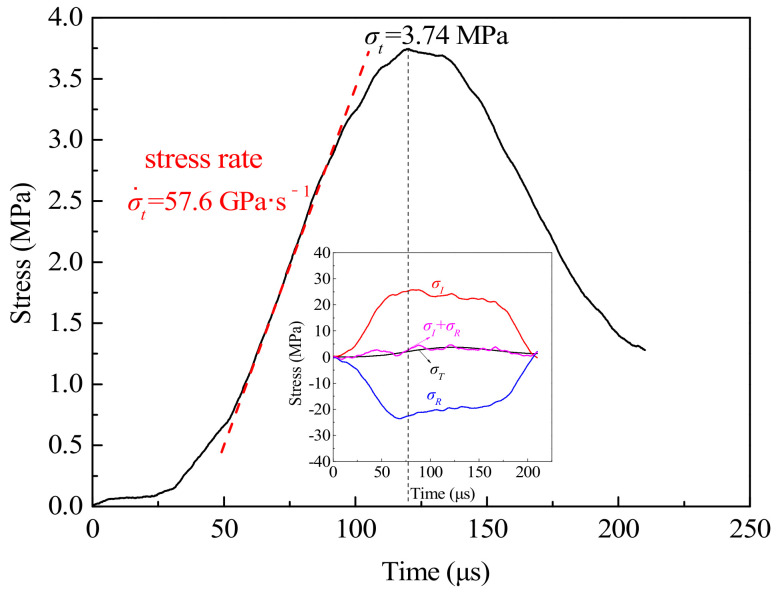
Stress rate selection method and stress balance verification.

Dynamic splitting tests of CFRC specimens with different carbon fiber contents (0.1, 0.2, and 0.3%) were carried out with the SHPB device under different impact pressures (0.15, 0.20, 0.25, and 0.30 MPa). Meanwhile, for the convenience of analysis and comparison, dynamic splitting tests were carried out for concrete specimens without carbon fiber at different impact pressures. In the dynamic splitting test, the stress rate is commonly used to characterize the rate effect of materials. The stress rate can be obtained according to Figure 7 [43], and the final stress rates ranged from 55 GPa·s^−1^ to 460 GPa·s^−1^. Under high stress rate, the increase of dynamic tensile strength is usually expressed by the dynamic increase factor (DIF), that is, the ratio of dynamic tensile strength to static tensile strength. The DIF can be obtained by Equation (3). Test results are shown in Table 5, and the analysis of the results will be carried out in the following chapters.

(3) $$DIF=\frac{f_{\mathrm{td}}}{f_{\mathrm{ts}}}$$

where *f*_td_ and *f*_ts_ are the dynamic and static tensile strength, respectively.

#### 3.2.1. Analysis on Typical Waveform Curve

**Figure 8:**
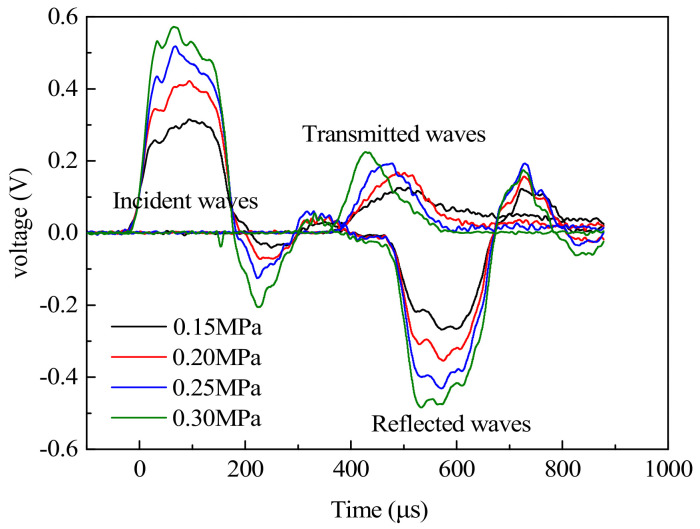
Typical voltage time−history curves.

The typical SHPB waveform curve was obtained through the experimental collection, as shown in Figure 8. It can be seen from Figure 8 that the action time of the incident waves, reflected waves, and the transmitted waves were relatively stable, and the signals of the incident wave and reflected wave were stronger than those of the transmitted wave. The main reason was that when the stress pulses propagated to the interface between the specimen and the incident bar, only a few pulses propagated to the transmitted bar to form the transmitted waves, and most of the pulses went back to the incident bar to form the reflected waves. The amplitudes of the incident waves, reflected waves, and transmission waves all increased with the increase of impact pressure, and the size of the reflected waves was positively correlated with the strain rate of concrete, that is, the strain rate of specimen increased with the increase of impact pressure. The second half of the reflected waves approached the platform, which showed that the strain rate closed to a constant, and the constant strain rate loading could be realized in the test. With the increase of the impact pressure, the peak value of the transmitted waves increased, and the time to reach the peak value was earlier.

From the analysis in Section 2.2, the test data were processed based on the stress equilibrium, and the stress equilibrium in the test must be verified to make the data effective and credible. According to the hypothesis of stress uniformity and one-dimensional stress wave theory, when the sum of the incident stress and the reflected stress is equal to the transmitted stress, the specimen is in a state of stress equilibrium. As shown in Figure 7, the sum of incident stress and reflected stress was close to transmitted stress, indicating that the specimen met the stress equilibrium condition and the test data processing was effective.

#### 3.2.2. Failure Process of Specimen

**Figure 9:**
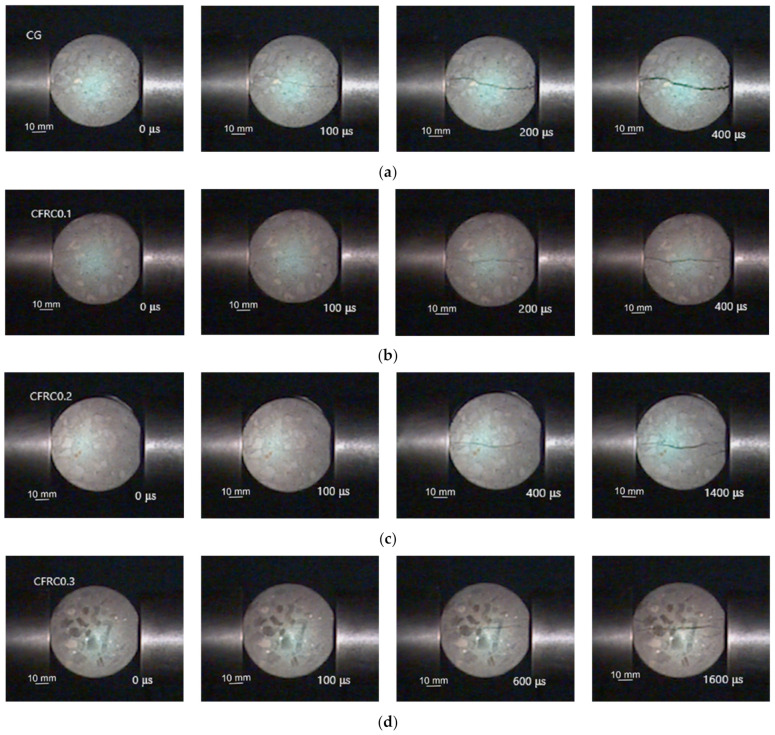
The dynamic failure process of the specimens, (**a**) CG, (**b**) CFRC0.1, (**c**) CFRC0.2, (**d**) CFRC0.3.

Combined with the high-speed camera technique, the failure patterns of CFRC under dynamic splitting tension were investigated by using the SHPB technique. To reflect the crack development process of the concrete specimen, the sampling frequency of 50,000 frames per second was adopted by the high-speed camera. The time difference between any two images taken with the high-speed camera was calculated as 20 μs. The crack propagation diagram of the concrete specimen under the same impact pressure is shown in Figure 9.

As shown in Figure 9, the failure process of CFRC0.1 was similar to that of CG, that is, the crack initiated in the center of the specimen and propagated along the loading direction to form the main crack. As the crack width increased, the specimen finally broke into two halves, showing the characteristic of sudden brittle failure. The failure process of CFRC0.2 was similar to that of CFRC0.3, due to the randomly distributed carbon fibers in the matrix effectively inhibiting the propagation of the crack, resulting in the crack growth rate slowing down, and many secondary micro-cracks were generated near the main crack. The addition of carbon fiber increased the number of cracks and delayed the failure time of specimens. With the increase of fiber content, this phenomenon was more distinct. CG and CFRC0.1 had many similarities in static and dynamic splitting tests, both of which showed sudden brittle failure. However, many matrix fracture fragments appeared during the dynamic failure process. Both CFRC0.2 and CFRC0.3 showed different ductile failure characteristics from static ones in dynamic tests. It can be seen that the deformation capacity of concrete could be improved by adding carbon fiber under dynamic loading. The main reason is that, when the concrete matrix under rapid loading at a high stress rate, the acceleration of the materials around the matrix will lead to additional clamping pressure on the fiber surface due to the inertial effect. The clamping pressure generated by the inertia effect on the fiber surface enhances the interfacial bond strength at the high stress rate, thus improving the deformation capacity of CFRC.

#### 3.2.3. Dynamic Stress Time-History of CFRC

**Figure 10:**
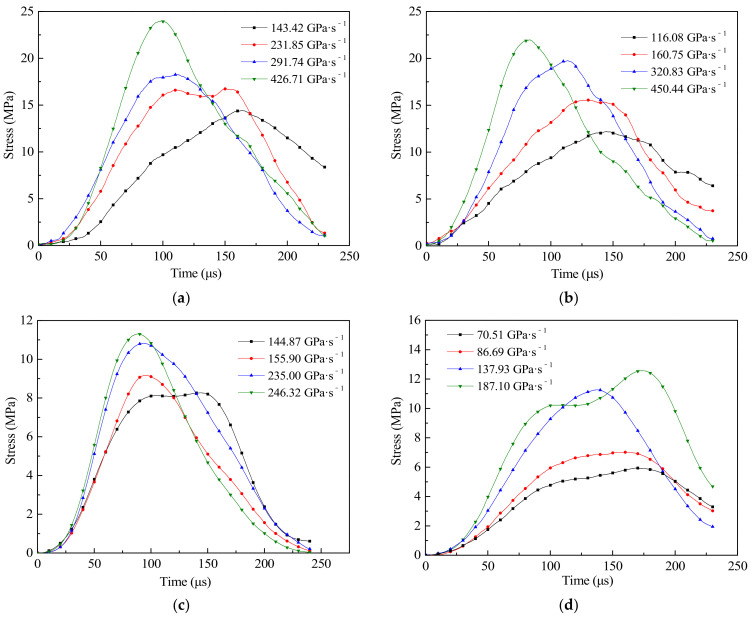
Stress-time curve of specimens with different types, (**a**) CG, (**b**) CFRC0.1, (**c**) CFRC0.2, (**d**) CFRC0.3.

To explain the change process in the dynamic tensile strength of CFRC more clearly, the test data were processed by Equation (2) to obtain the stress time-history curves of specimens with different carbon fiber content, as shown in Figure 10.

From Figure 10, the dynamic tensile stress of CG and CFRC had similar rules with the change of time, and the stress time-history curves remained unchanged with the increase of carbon content. Compared with the static tests, the specimens had higher tensile properties at high stress rates, showing a significant rate effect. The rate sensitivity of concrete can be attributed to the crack propagation mode and the movement of free water in the matrix [44,45]. However, the effect of water on the rate sensitivity may be much less than that of crack propagation, because the crack propagation rate increases at high stress rates, so crack propagation plays a major role in the rate effect of materials. Under static loading, the micro-cracks of CFRC had enough time to expand to the macro-cracks, which led to the final failure of the specimens. In general, the energy required for crack formation is greater than that required for crack development, and the failure of concrete specimens often extend along with the aggregate–matrix interface of the lowest strength [46]. However, under dynamic loading, only a few micro-cracks attempted to expand to macro-cracks before the final failure occurred, while more micro-cracks nucleated and grew. Under high stress rate, there was not enough time for internal cracks to develop, and the tensile strength of concrete increased with the increase of stress rate.

#### 3.2.4. Dynamic Tensile Strength of CFRC

**Figure 11:**
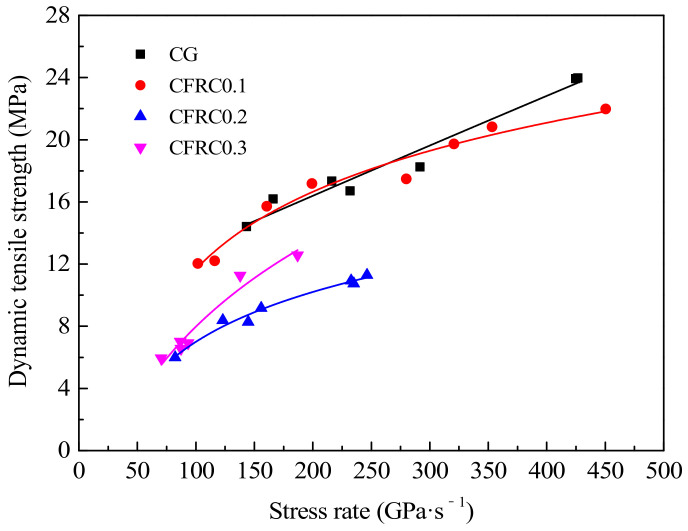
Relationship between dynamic tensile strength and stress rate.

As shown in Figure 11, the effect of stress rate on tensile strength was significant, but the influence of carbon fiber content on dynamic tensile strength was completely different from that of static strength. Under static loading, the tensile strength increased significantly with the increase of carbon fiber content, although the amplitude gradually decreased. Under dynamic loading, the tensile strength of CFRC0.1, CFRC0.2, and CFRC0.3 were lower than CG, indicating that the addition of carbon fiber reduced the dynamic tensile strength of concrete, but the tensile strength of CFRC0.3 was greater than that of CFRC0.2. Besides, an interesting phenomenon is observed in Figure 11. Under the low stress rate, the growth rate in the tensile strength of CFRC was higher than that of the high stress rate. With the increase of stress rate, the growth rate of tensile stress gradually decreased. This also indirectly indicated that the rate enhancement mechanism of CFRC was different from that of ordinary concrete. The reason may be that CFRC underwent three stages of initial crack generation, crack propagation and new crack generation, and fiber fracture or pull-out under the action in the dynamic load of low stress rate. With the increase of stress rate, the time interval between new cracks and carbon fiber fracture or pull-out became shorter or occurred simultaneously. The energy consumption of specimens in finite time mainly depended on the generation of new cracks [14]. However, the initial crack strength of CFRC increased due to the rate effect of the material, the tensile strength was improved slightly by the subsequent generation of new cracks and fiber pull-out or fracture, with the result that the strengthening effect of CFRC was not obvious at high stress rate. The generation of the initial crack was related to the tensile strength of the concrete matrix, so the tensile stress at high stress rate mainly depended on the strength of the CFRC matrix [47].

**Figure 12:**
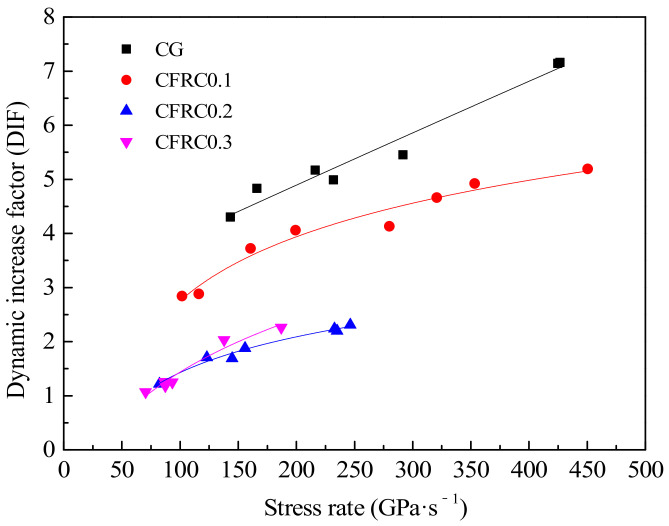
Relationship between the DIF and stress rate.

**Figure 13:**
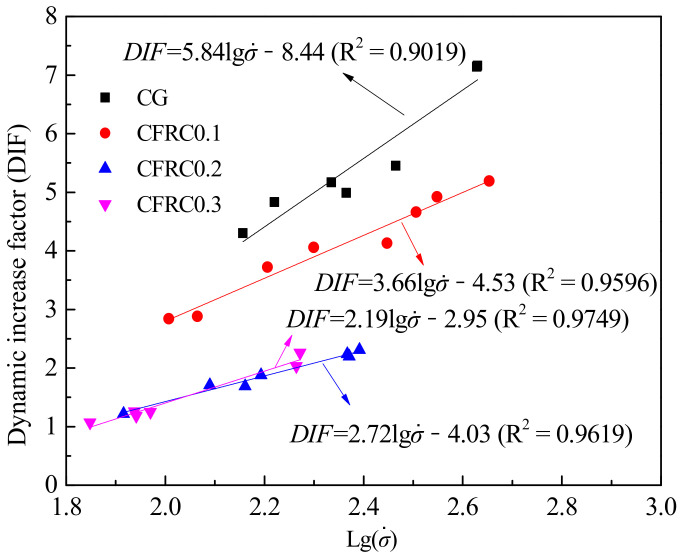
The fitting lines and fitting equations between the DIF and the logarithm of stress rate.

The distribution of DIF values under different stress rates is shown in Figure 12. When the volume content of carbon fiber was between 0 and 0.2%, DIF values of different carbon fiber contents were greatly different, and it decreased significantly with the increase of carbon fiber content. It is mainly because the concrete with high static strength usually shows a low DIF value [14], while CFRC has higher static strength, so it has a lower DIF value. However, it is worth noting that DIF values of CFRC0.3 were slightly higher than those of CFRC0.2. As can be seen from the variation trend of the fitting curves in Figure 12, with the increase of stress rate, DIF gradually increased and the growth rate gradually decreased, which was similar to the growing trend in Figure 9 and close to the growth of the logarithmic function. Long [48] suggested Equation (4) to represent the relationship between DIF and the logarithm of strain rate. In this paper, the rate effect of concrete material was characterized by the stress rate instead of the strain rate. The fitting lines and fitting equations between DIF and logarithm of stress rate are shown in Figure 13.

(4) $$DIF=a\cdot \mathrm{lg}\dot{\varepsilon }+b$$

From Figure 13, both CG and CFRC showed a similar rate sensitivity trend, DIF values increased linearly with the increase of the logarithm of the stress rate. The slope of the fitting curve in the control group was 5.84, while those of CFRC0.1, CFRC0.2, and CFRC0.3 were 3.66, 2.19, and 2.72, respectively. It showed that the control group specimens had higher rate sensitivity than CFRC, which was similar to the steel fiber reinforced concrete. Many researchers [49,50,51] have reported that concrete with higher fiber content has lower rate sensitivity. The reason for the low rate sensitivity in CFRC specimens may be that the carbon fiber in the crack path reduced the crack growth rate. However, the rate sensitivity of CFRC0.3 was slightly higher than that of CFRC0.2, indicating that the rate sensitivity does not decrease with the increase of carbon fiber content alone. It may be related to the bonding strength between the CFRC matrix and fiber and the mechanism of carbon fiber fracture or pull-out.

## 4. Discussion

Through the special research on CFRC, although some valuable research results were obtained, there are still some phenomena and problems that need to be further analyzed and discussed.

Previous studies on the influence of carbon fiber content and length on the mechanical properties of concrete [18,26,52] have found that as the content of carbon fiber increases, the fluidity of slurry decreases continuously, its dispersion becomes more difficult, and the instability of complex properties increases. CFRC has good splitting strength when the volume content of carbon fiber is less than 1%. Therefore, this paper only took carbon fiber concrete with the length of 20 mm and the content of 0.1, 0.2, and 0.3% as the research object to discuss its tensile properties under different loading modes. The experimental variables were insufficient, and further research is needed.

**Figure 14:**
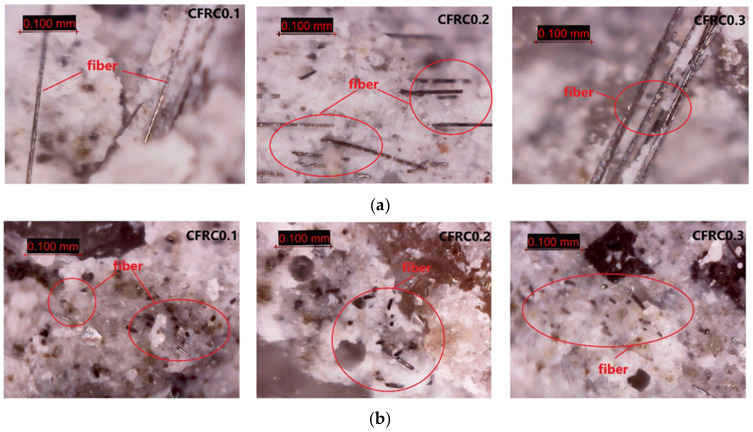
Mesoscopic fracture images of specimens under (**a**) static loading and (**b**) dynamic loading.

According to the above analysis of the test results of CFRC, under static loading, the tensile strength increased significantly with the increase of carbon fiber content. Under impact loading, the dynamic tensile strength of concrete decreased with the addition of carbon fiber, which was different from the static test results. To explore the reasons for the difference between dynamic and static mechanical properties of concrete, the digital electron microscope with an amplification factor of 1000 was used to observe the fracture surface of CFRC. The observation results of the microscopic structure are shown in Figure 14.

From Figure 14, although some carbon fibers were distributed together, they did not form clusters or coils; the carbon fibers had good dispersion. Under static loading, most of the carbon fibers were pulled out and played an obvious bridging role at the crack. Under dynamic loading, there were some crystal fragments on the fracture surface. Most of the fibers were embedded in the matrix and only a small part was exposed, indicating that large quantities of fibers were broken during the failure. The addition of carbon fiber had both strengthening and weakening effects on the properties of concrete. On the one hand, the high strength of carbon fiber itself consumed energy in the process of pulling out or fracture, which improved the mechanical properties of CFRC. On the other hand, the addition of carbon fiber caused some bubbles and pores in the material and generated some weak surfaces between the fiber and the matrix, which would damage the integrity of the concrete matrix.

The phenomenon of the mesoscopic structure in Figure 14 was consistent with the measured results of mechanical properties. Under static loading, the crack propagation was dominant, and the fiber could play a bridging role to restrain the crack propagation. Besides, the fiber had enough time to be pulled out. Before being pulled out, it could bear the continuous load and consume a lot of energy, so as to improve the tensile strength of CFRC. Under dynamic loading, the interior pores and micro-cracks in the concrete were rapidly connected, the cracks did not have enough time to develop, and the carbon fiber could not perform its full function because of the quick fracture. Moreover, the tensile stress under the high stress rate mainly depended on the strength of the concrete matrix. The integrity of the matrix was destroyed with the addition of carbon fiber, and the weakening effect was dominant. Therefore, the dynamic tensile strength of CFRC was lower than that of ordinary concrete.

The dynamic tensile strength of carbon fiber with a content of 0.3% was higher than that with 0.2%, which means that the dynamic tensile strength has a rising phenomenon as the fiber content continues to increase. The reason may be that the content of carbon fiber in CFRC0.3 is relatively high. Under impact loading, some fibers are not directly fractured but broken after being pulled out, which improves the mechanical properties. In subsequent research, the reason for dynamic tensile strength rebound can be further studied in view of the high content of carbon fiber, and the strength variation law of CFRC under impact load can be found out, which can provide more reliable reference for engineering applications. However, there were inevitable errors in the specimen preparation and testing system. The main influencing factors included the dispersion of carbon fiber in concrete, the stability of impact pressure and the accuracy of the acquisition system. Therefore, the failure mode and tensile properties of CFRC specimens need to be further studied on this basis.

## 5. Conclusions

In this paper, the dynamic splitting tensile tests of CFRC with different contents were carried out by using the SHPB test device, and its dynamic tensile strength, DIF, and damage characteristics were analyzed and researched. The differences between dynamic and static mechanical properties of CFRC were discussed. The following conclusions were drawn:

1. With the increase of fiber content, the static tensile strength of CFRC increases obviously, but the increased amplitude tends to decrease. From the perspective of failure morphology, both CG and CFRC specimens show the characteristics of brittle failure. Under static load, carbon fiber does not significantly improve the ductility of concrete specimens.
2. When the fiber content is constant, the dynamic tensile strength and DIF both increase with the increase of stress rate, which has an obvious rate effect, but the growth rate becomes slower. Contrary to static loading, the addition of carbon fiber makes the dynamic tensile strength and DIF of CFRC lower than CG, but the failure form shows certain ductility characteristics.
3. CG and CFRC specimens show a similar rate sensitivity trend, and the DIF value of the specimen increases linearly with the increase of the logarithm of the stress rate. Besides, CG specimens have higher rate sensitivity than CFRC, and the rate sensitivity does not decrease only with the increase of carbon fiber content.
4. The static tensile strength of CFRC is significantly improved, and the tension–compression ratio of concrete is increased, which is of great significance to concrete. This improvement reduces the risk of unpredicted damage to concrete structures, which means that it has a wide application prospect. But the addition of carbon fiber has a negative effect on dynamic tensile strength. Obviously, the content of carbon fiber should be selected according to the requirements of the application field, so that the compressive strength and tensile strength/impact resistance can be balanced.

## Figures and Tables

**Table 1:** Chemical composition and geometric characteristics of cement and silica fume [31].

Component	Density (kg·m^−^^3^)	BET (m^2^·g^−1^)	Chemical Composition (wt.%) (XRF)					
			CaO	SiO_2_	Al_2_O_3_	Fe_2_O_3_	SO_3_	MgO
Cement	1910	1.477	31.31	1.94	0.9	0.23	43.49	0.29
Silica fume	310	23.7	0.8	97	0.6	0.1	1.0	-

**Table 2:** Chemical composition of tap water (mg/L).

Fluoride	Nitrate	Chlorite	Chloride	Sulfate	Mn	Cu	Zn	Al	Fe	Pb	pH
0.38	8.81	0.15	70.8	31.2	<0.05	<0.05	<0.05	<0.007	<0.05	<0.0025	7.76

**Table 3:** Parameters of carbon fiber.

Tensile Strength (MPa)	Density (kg·m^−3^)	Tensile Modulus (GPa)	Elongation (%)	Length (mm)
3530	1760	230	1.5	20

**Table 4:** Mix proportions of concrete (kg·m^−3^).

Specimen Type	Cement	Water	Fine Aggregate	Coarse Aggregate	Coal Ash	Silica Fume	Water Reducing Agent	Rubber	Carbon Fiber
CG	385.9	154.36	699	1140	45.4	22.7	7.8	9.08	0
CFRC0.1	385.9	154.36	699	1140	45.4	22.7	7.8	9.08	1.8
CFRC0.2	385.9	154.36	699	1140	45.4	22.7	7.8	9.08	3.6
CFRC0.3	385.9	154.36	699	1140	45.4	22.7	7.8	9.08	5.4

**Table 5:** Mechanical properties of dynamic splitting tests.

Specimen Number	Carbon Fiber Content (%)	Stress Rate (GPa·s^−1^)	Dynamic Tensile Strength (MPa)	Static Tensile Strength (MPa)	Dynamic In-Crease Factor (DIF)
CG-1	0	143.42	14.41	3.35	4.30
CG-2		166.11	16.19	3.35	4.83
CG-3		216.18	17.33	3.35	5.17
CG-4		231.85	16.71	3.35	4.99
CG-5		291.74	18.25	3.35	5.45
CG-6		424.88	23.93	3.35	7.14
CG-7		426.71	23.98	3.35	7.16
CFRC0.1-1	0.1	101.69	12.03	4.23	2.84
CFRC0.1-2		116.08	12.20	4.23	2.88
CFRC0.1-3		160.75	15.72	4.23	3.72
CFRC0.1-4		199.27	17.17	4.23	4.06
CFRC0.1-5		280.00	17.47	4.23	4.13
CFRC0.1-6		320.83	19.73	4.23	4.66
CFRC0.1-7		353.33	20.82	4.23	4.92
CFRC0.1-8		450.44	21.97	4.23	5.19
CFRC0.2-1	0.2	82.38	5.99	4.89	1.22
CFRC0.2-2		123.02	8.38	4.89	1.71
CFRC0.2-3		144.87	8.27	4.89	1.69
CFRC0.2-4		155.9	9.17	4.89	1.88
CFRC0.2-5		232.84	10.93	4.89	2.24
CFRC0.2-6		235.00	10.75	4.89	2.20
CFRC0.2-7		246.32	11.30	4.89	2.31
CFRC0.3-1	0.3	70.51	5.94	5.55	1.07
CFRC0.3-2		86.69	7.01	5.55	1.26
CFRC0.3-3		87.41	6.56	5.55	1.18
CFRC0.3-4		93.33	6.92	5.55	1.25
CFRC0.3-5		137.93	11.26	5.55	2.03
CFRC0.3-6		187.10	12.57	5.55	2.26

## Data Availability

The raw/processed data required to reproduce these findings cannot be shared at this time as the data also form part of an ongoing study.
